# Health‐related quality of life in patients with alopecia areata: Results of a Japanese survey with norm‐based comparisons

**DOI:** 10.1111/1346-8138.16364

**Published:** 2022-03-28

**Authors:** Taisuke Ito, Kazumasa Kamei, Akira Yuasa, Fumihiro Matsumoto, Yayoi Hoshi, Masafumi Okada, Shinichi Noto

**Affiliations:** ^1^ Department of Dermatology Hamamatsu University School of Medicine Hamamatsu Japan; ^2^ Inflammation and Immunology Medical Affairs Pfizer Japan Inc. Tokyo Japan; ^3^ Health and Value Pfizer Japan Inc. Tokyo Japan; ^4^ Real‐World Evidence Solutions & HEOR IQVIA Solutions Japan K.K. Tokyo Japan; ^5^ Department of Rehabilitation Niigata University of Health and Welfare Niigata Japan

**Keywords:** alopecia areata, cross‐sectional studies, mental health, patient reported outcome measures, quality of life

## Abstract

Alopecia areata (AA) is a non‐scarring hair loss disorder affecting approximately 2% of the global population. AA is reported to have a significant negative impact on the emotional and psychological well‐being of the patients. This study aimed to evaluate the health‐related quality of life (HRQoL) of Japanese patients with AA in comparison to the Japanese population norms (national standard values for Japanese) using Short Form Health Survey 36 Item Version 2.0 (SF‐36v2). The study also aimed to access the negative effect of AA on patients’ daily lives and the proportion of patients having anxiety and/or depression. This cross‐sectional, non‐interventional web‐based survey study included 400 participants aged 17–84 years currently suffering from medically diagnosed AA. The assessment tools integrated in the online questionnaire included SF‐36v2, the Dermatology Life Quality Index (DLQI), and the Hospital Anxiety and Depression Scale (HADS). All outcome measures from the tools were evaluated across the study population. SF‐36v2 subscale scores for patients with AA revealed lower scores specifically for mental health (45.7 ± 10.1 points), social functioning (45.8 ± 10.9 points), vitality (46.2 ± 9.8 points), and role emotional (46.9 ± 11.6 points) as compared to the Japanese population norms of 50 ± 10 points each. The DLQI questionnaire‐based analysis indicated that 32.1% of respondents showed a moderate to extremely large effect on their lives; and HADS‐A (anxiety) and HADS‐D (depression) scores categorized 46.0% and 41.8% respondents as doubtful‐to‐definite cases, respectively. Multivariate linear regression revealed that hair loss range, age, comorbidities, and depression significantly worsened DLQI scores. In conclusion, the results of this survey demonstrated that a significant decrease in the HRQoL scores was observed in Japanese patients with AA in comparison with the national norms. Hence, emphasis on mental health is crucial for AA management.

## 1 INTRODUCTION

Alopecia areata (AA) is an autoimmune‐mediated nonscarring hair loss disorder^1^, ^2^ with a reported global lifetime risk of approximately 2%.^3^ Approximately 14–25% of patients with AA experiencing alopecia totalis and universalis have a poor recovery rate (<10%).^4^ Although AA is not a debilitating condition, it has significant impact on the psychosocial condition and health‐related quality of life (HRQoL) of patients.^5^, ^6^, ^7^

Studies have highlighted that patients with AA with both clinically noticeable and negligible hair loss have a significantly decreased HRQoL.^6^, ^8^ Hair loss may significantly impair individuals’ self‐esteem and emotional harmony.^9^ Social stigmatization or even its perception in patients may result in shy, cautious, aggressive, retreating, evasive, or defensive behavior.^4^ Lack of awareness of AA is another unmet need prevailing among individuals experiencing hair loss and they often misconstrue hair loss as just cosmetic and are negligent about it; however, this chronic disorder can lead to diverse comorbidities which may further influence HRQoL.^10^ Multiple comorbid conditions associated with AA include atopic, metabolic, rheumatoid, thyroid, and psychiatric diseases.^11^ Numerous studies have reported a greater risk of psychiatric disorders in patients with AA.^7^, ^12^, ^13^, ^14^ Since the physical, psychological, and social well‐being of patients with AA are affected to a great extent, an increased incidence of depression and anxiety are expected which may result in higher suicidal risk.^6^, ^12^ A study conducted in 2021 on an Israeli registry with 41 055 patients with AA corroborated that anxiety and depression were the positively associated disorders with AA while schizophrenia and bipolar disorder were negatively or not associated with AA.^15^ According to the British Association of Dermatologists’ guidelines, psychological support is recommended as an essential intervention in AA.^4^ Hence, it is critical to assess the HRQoL of patients for better management of AA.

Several studies have examined the impact of AA on HRQoL worldwide through various clinical outcome assessment tools.^6^, ^8^ The Short Form Health Survey 36 Item Version 2.0 (SF‐36v2) is one of the most widely used instruments for evaluating HRQoL.^16^, ^17^ It is a multipurpose, short‐form health survey with only 36 questions; and it is a generic measure as opposed to one that targets specific age, disease, or treatment groups. The Dermatology Life Quality Index (DLQI)^18^ is a tool recommended by the European Academy of Dermatology and Venereology Task Force on Quality of Life and Patient Oriented Outcomes for evaluating HRQoL for patients with AA.^19^ A meta‐analysis on HRQoL instruments has identified the SF‐36v2 and DLQI tools to be the most common measures used in patients with AA.^6^ Additionally, tools like the Hospital Anxiety and Depression Scale (HADS) have been used to report positive association of AA with anxiety and depression.^13^

Although there have been several studies globally that have acknowledged HRQoL importance in patients with AA, there are noticeable limitations. Most of the studies have smaller patient populations and lack control cohorts.^7^ Recently, normative scoring has been developed for the US population,^20^ but there is very limited clinical evidence comparing SF‐36v2 data against normative scores.^6^ Further, evidence from multivariate analysis adjusting for possible confounders is limited for HRQoL studies in patients with AA.^21^, ^22^ Finally, no evidence with SF‐36v2 has been reported for Japanese patients with AA. Hence, this study aims to address the above limitations.

The primary objective of this study was to evaluate HRQoL in Japanese patients with AA in comparison to Japanese population norms (national standard values for Japanese) using the SF‐36v2 measurement tool. Secondary objectives included: (i) evaluation of the impact of AA on HRQoL using DLQI and determination of the proportion (%) of depression and anxiety in patients with AA using HADS; and (ii) evaluation of factors (e.g., hair loss range, comorbidities) that may substantially impact HRQoL using multivariate analysis. This is the first‐of‐its‐kind study that aimed to explore correlations among SF‐36v2, DLQI, and HADS scores.

## 2 METHODS

### 2.1 Study design

This is a cross‐sectional, non‐interventional study that evaluated the impact on HRQoL of Japanese patients with AA. All alopecia patients who were registered in the Rakuten Insight Disease Panel (Rakuten Insight; https://rd.insight.rakuten.net/l/310391/2020‐09‐07/w7bk3r) were invited to participate in this study. The patients who responded signed the informed consent form and completed a web‐based screening survey to identify eligible participants aged 17–84 years and currently suffering from medically diagnosed AA. Participants who had not visited a hospital for AA for the past 1 year, and those with androgenetic alopecia or other forms of alopecia, were excluded from the study. All eligible participants were invited to complete another web‐based survey including sociodemographic and HRQoL questionnaires. The survey was conducted and the data were collected during 12–17 March 2021. The study was approved by the Ethical Review Board of the Specified Non‐profit Organization MINS (200245) (http://www.npo‐mins.com/) and was conducted in accordance with the Declaration of Helsinki, Good Pharmacoepidemiology Practices, and Ethical Guidelines for Medical and Health Research Involving Human Subjects (Ministry of Health, Labor, and Welfare [MHLW], Japan).

### 2.2 Outcome assessments

The outcomes evaluated for AA participants were based on data collected using the SF‐36v2, DLQI, and HADS instruments. At the beginning of the study, a demographic and AA‐specific questionnaire was developed that included questions related to sex, age, employment status, relationship status, comorbidities, hair loss range, type of AA diagnosed and its duration, treatment, and so forth. The appropriateness of the questionnaire was reviewed and validated by three expert clinicians from the Japanese Dermatological Association. SF‐36v2, a coherent HRQoL measurement tool, includes 36 questions that assess eight subscales: physical functioning (PF), role physical (RP), bodily pain (BP), general health (GH), vitality (VT), social functioning (SF), role emotional (RE), and mental health (MH);^23^ and two component scores: the physical component summary (PCS) and the mental component summary (MCS).^23^ The original scoring method uses scores ranging from 0 to 100, with higher scores suggesting better HRQoL.^23^ The DLQI includes 10 questions related to factors affecting skin disorders and scores range from 0 to 30.^18^ DLQI has been categorized into band 0 (score 0–1), band 1 (score 2–5), band 2 (score 6–10), band 3 (score 11–20), and band 4 (score 21–30) corresponding to no effect, small effect, moderate effect, very large effect, and extremely large effect, respectively.^24^ The HADS questionnaire is a 14‐item scale used to capture any mental disparities experienced by the participants.^25^ The HADS is graded from 0 to 21 points and scores of 0–7, 8–10, and 11–21 are defined as non, doubtful, and definite, respectively.^25^

### 2.3 Statistical analysis

The sample size was estimated with the assumption to obtain 50% of the event proportion with 10% width as a 95% confidence interval. An estimated required sample size of 384 and 400 was set as the target by multiplying by 1.05 in consideration of dropouts. Descriptive statistics (n, mean, median, standard deviation [SD], range, min and max for continuous variables; percentage for categorical variables) were used to summarize the sociodemographic and clinical characteristics. The scores for SF‐36v2 were interpreted using norm‐based scoring (NBS), which is the deviation scores from the Japanese population norms (national standard values for Japanese). Japanese population norms used in this study were the average data from a survey conducted in 2017.^26^ The DLQI and HADS questionnaires’ outcomes were evaluated and presented as percentages (number of patients) per defined category. Subpopulation analysis was performed using the descriptive statistics mentioned above for specific subgroups (sex, age, hair loss range, AA type, disease duration, relapse experience, comorbidities, current treatment, and wig usage) on all subscales of the SF‐36v2, DLQI, and HADS questionnaires. Correlation coefficients were calculated to evaluate correlations among the SF‐36v2, DLQI, and HADS scores. Further, multivariate linear regression analysis was performed to evaluate the effects of the characteristics in specific subgroups on the SF‐36v2 and DLQI scores.

## 3 RESULTS

### 3.1 Respondents’ disposition, demographic and clinical characteristics

A total of 32 257 subjects (the total unique number of subjects who registered as an alopecia panel between 2013 and 2021 at the time of the survey) were invited to participate in this study of which 16 522 respondents gave consent. While 537 participants met the inclusion criteria for AA defined in this study, 400 completed the entire survey. The baseline characteristics of respondents are presented in Table 1. The mean age ± SD of the respondents was 42.9 ± 11.6 years, and the majority of respondents were female (67.3%). Clinically, 83.5% of the respondents reported a hair loss range of less than 25% while 8.3% each of respondents reported hair loss ranges of 25–49% and 50–100%. Approximately 38.8% of the respondents reported to have some comorbidities such as atopic dermatitis.

**TABLE 1:** Demographic and clinical characteristics

Variables	n (%)
Sex	
Male	131 (32.8)
Female	269 (67.3)
Age	
17–19	0 (0.0)
20–29	62 (15.5)
30–39	98 (24.5)
40–49	129 (32.3)
50–84	111 (27.8)
Hair loss range	
<25%	334 (83.5)
25–49%	33 (8.3)
≥50%	33 (8.3)
Alopecia type	
Single	231 (57.8)
Multi	136 (34.0)
Ophiasis	14 (3.5)
Others^a^	19 (4.8)
Comorbidities	
With^b^	155 (38.8)
Without	245 (61.3)
Disease duration	
0–11 months	180 (45.0)
1–4 years	112 (28.0)
≥5 years	108 (27.0)
Relapse experience	
Never	125 (31.3)
≥1 time	275 (68.8)
Current treatment at hospital	
Yes	275 (68.8)
No	125 (31.3)
Current usage of wigs	
Yes	22 (5.5)
No	378 (94.5)

Abbreviation: n, number of participants in the category.

^a^ Population in others section included both alopecia totalis and universalis.

^b^ Atopic dermatitis (n = 62), vitiligo vulgaris (n = 5), thyroid disease (n = 11), type 1 diabetes (n = 6), myasthenia gravis (n = 0), systematic lupus erythematosus (n = 2), rheumatoid arthritis (n = 2), psoriasis (n = 8), inflammatory bowel disease (n = 2), mineral deficiency (n = 3), anxiety (n = 39), depression (n = 26), other (n = 45).

### 3.2 Outcome assessments

#### 3.2.1 Analysis of SF‐36v2

**FIGURE 1:**
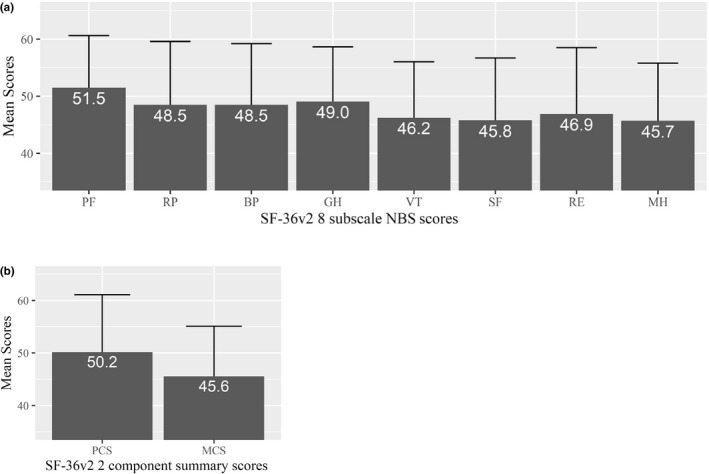
SF‐36v2 subscale scores (a) and component summary scores (b) based on norm‐based scoring. Error bars indicate standard deviation. Norm‐based scoring (NBS) is calculated using scores from 0 to 100, assuming Japanese population norms as 50 ± 10 points each. BP, bodily pain; GH, general health; MCS, mental component summary; MH, mental health; PCS, physical component summary; PF, physical functioning; RE, role emotional; RP, role physical; SF, social functioning; SF‐36v2, Short Form Health Survey 36 Item version 2.0; VT, vitality

The primary analysis of the eight subscales assessed with SF‐36v2 concluded that patients with AA have lower HRQoL scores especially for MH (45.7 ± 10.1 points), SF (45.8 ± 10.9 points), VT (46.2 ± 9.8 points), and RE (46.9 ± 11.6 points) measures in comparison with the Japanese population norms (50 ± 10 points) (Figure 1a). Based on the two‐component summary scores, MCS (45.6 ± 9.6 points) was lower but PCS (50.2 ± 10.9 points) was similar in comparison with Japanese population norms (50 ± 10 points) (Figure 1b). SF‐36v2 results scored with 0–100 normative points are shown in Figure S1. Based on the subgroup analysis of SF‐36v2 NBS performed for sex characteristics, similar scores were observed between males and females for all eight subscales (Table S1).

#### 3.2.2 Analysis of DLQI

Based on the DLQI scores, the study population was categorized with 32.1% of the patients with AA reporting moderate (17.5%), very large (13.3%), or extremely (1.3%) large effects on their lives (Table 2). The mean (SD) DLQI score was 4.8 points (5.2).

**TABLE 2:** DLQI and HADS outcomes

Scale	n (%)
DLQI	
0–1 (no effect)	131 (32.8)
2–5 (small effect)	141 (35.3)
6–10 (moderate effect)	70 (17.5)
11–20 (very large effect)	53 (13.3)
21–30 (extremely large effect)	5 (1.3)
HADS‐A	
0–7 (non)	216 (54.0)
8–10 (doubtful)	84 (21.0)
11–20 (definite)	100 (25.0)
HADS‐D	
0–7 (non)	233 (58.3)
8–10 (doubtful)	89 (22.3)
11–20 (definite)	78 (19.5)

Abbreviations: DLQI, Dermatology Life Quality Index; HADS‐A, Hospital Anxiety and Depression Scale – Anxiety; HADS‐D, Hospital Anxiety and Depression Scale – Depression; n, number of participants in the category.

**FIGURE 2:**
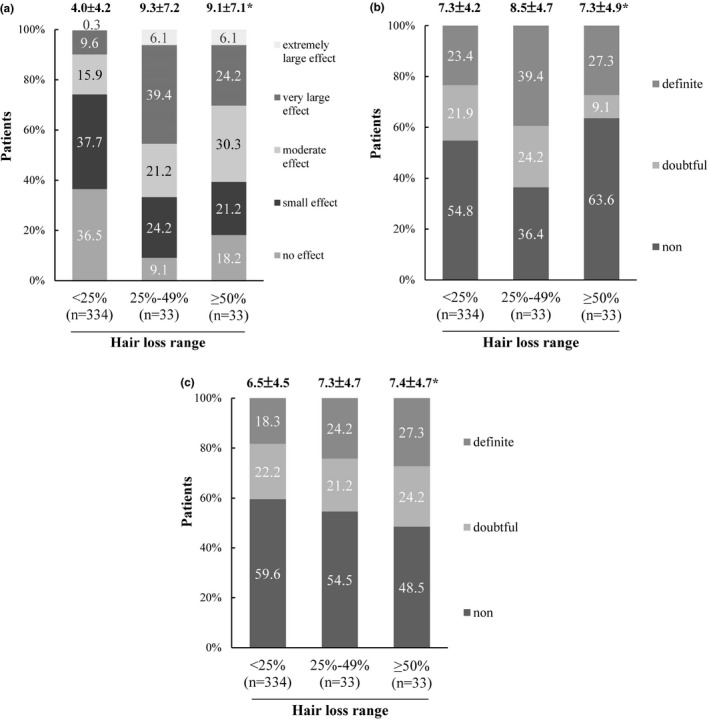
Subgroup analysis of DLQI (a), HADS‐A (b), and HADS‐D (c) by hair loss range. Panel (a) shows the percentage of patients who were classified into five groups using the Dermatology Life Quality Index (DLQI) as follows: no effect at all (0–1 points), small effect (2–5 points), moderate effect (6–10 points), very large effect (11–20 points), and extremely large effect (21–30 points) on the patient’s life. Panels (b) and (c) show the percentage of patients who were classified into three groups using Hospital Anxiety and Depression Scale – Anxiety (HADS‐A) and – Depression (HADS‐D), respectively, as follows: non‐cases (0–7 points), doubtful cases (8–10 points), and definite cases (11–21 points). *Mean ± standard deviation (in points)

In the hair loss range subgroup analysis for DLQI scores, the proportion of respondents who reported moderate to extremely large effects on their lives is higher in the groups with 25–49% and 50–100% hair loss than those with less than 25% hair loss (66.7% and 60.6%, respectively, vs 25.7%) (Figure 2a).

#### 3.2.3 Analysis of HADS

Based on HADS‐A (anxiety), 54.0% of respondents reported to have no anxiety while 21.0% were categorized as doubtful cases and 25.0% as definite cases (Table 2). According to HADS‐D (depression), 58.3% of respondents reported to have no depression while 22.3% and 19.5% were categorized as doubtful and definite cases, respectively (Table 2). In the overall study population, the mean scores were 7.4 ± 4.3 for HADS‐A and 6.6 ± 4.5 for HADS‐D, respectively, and 55.3% (n = 221) were categorized into doubtful or definite cases of physiological distress, namely anxiety and/or depression.

For HADS‐A, the mean score of respondents was higher in the group with 25–49% hair loss than in groups with less than 25% and 50–100% hair loss (8.5 ± 4.7 vs 7.3 ± 4.2 or 7.3 ± 4.9, respectively) (Figure 2b). For HADS‐D, the hair loss range dependency observed was unlike those for HADS‐A outcomes, with the mean scores of patients being similar at 6.5 ± 4.5, 7.3 ± 4.7, and 7.4 ± 4.7 for hair loss ranges of less than 25%, 25–49%, and 50% or more, respectively (Figure 2c).

#### 3.2.4 Outcome correlations

Results of the correlation analysis between SF‐36v2 and HADS indicated that the MH subscale is the most correlated SF‐36v2 subscale against HADS‐A (correlation coefficient, −0.70) and HADS‐D (−0.69) (*p* < 0.01 for both) (Table 3). The correlation coefficients of the VT and RE subscales against HADS‐A were −0.55 and −0.58, respectively, and against HADS‐D were −0.64 and −0.60, respectively (*p* < 0.01 for all). The correlation coefficients for the other five subscales were −0.43 to −0.53 for HADS‐A and −0.41 to −0.58 for HADS‐D. MH, RE, and VT were the top three subscales that have high correlation with HADS‐A.

**TABLE 3:** Correlations between SF‐36v2, DLQI, and HADS

Assessment tool/components	SF‐36v2								DLQI	HADS	
	PF	RP	BP	GH	VT	SF	RE	MH		HADS‐A	HADS‐D
SF‐36v2											
PF											
RP	0.56**										
BP	0.37**	0.53**									
GH	0.39**	0.53**	0.51**								
VT	0.28**	0.46**	0.43**	0.62**							
SF	0.33**	0.53**	0.39**	0.49**	0.43**						
RE	0.49**	0.83**	0.48**	0.55**	0.52**	0.54**					
MH	0.35**	0.52**	0.42**	0.63**	0.74**	0.52**	0.59**				
DLQI	−0.34**	−0.47**	−0.27**	−0.30**	−0.28**	−0.43**	−0.48**	−0.41**			
HADS											
HADS‐A	−0.43**	−0.53**	−0.43**	−0.53**	−0.55**	−0.52**	−0.58**	−0.70**	0.42**		
HADS‐D	−0.44**	−0.56**	−0.41**	−0.58**	−0.64**	−0.50**	−0.60**	−0.69**	0.47**	0.72**	

*Note:* *p < 0.05; **p < 0.01.

Abbreviations: BP, bodily pain; DLQI, Dermatology Life Quality Index; GH, general health; HADS‐A, Hospital Anxiety and Depression Scale – Anxiety; HADS‐D, Hospital Anxiety and Depression Scale – Depression; MH, mental health; PF, physical functioning; RE, role emotional; RP, role physical; SF‐36v2, Short Form Health Survey 36 Item Version 2.0; SF, social functioning; VT, vitality.

#### 3.2.5 Multivariate linear regression

A multiple linear regression analysis for SF‐36v2 revealed that higher HADS‐A and HADS‐D scores significantly decreased all eight subscales (*p* < 0.01) (Table 4). The standardized regression coefficients ranged from −0.155 to −0.419 for HADS‐A and from −0.204 to −0.517 for HADS‐D. The presence of any comorbidity also significantly decreased seven subscales (−0.068 to −0.179; *p* < 0.01) except for BP (−0.069; *p* = 0.127).

**TABLE 4:** Multivariate linear regression

Variable	SF‐36v2								DLQI
	PF	RP	BP	GH	VT	SF	RE	MH	
Sex									
Female	0.038	−0.036	−0.121*	−0.056	−0.106*	−0.049	−0.075	−0.062	−0.040
Age	−0.150**	−0.004	−0.211**	−0.073	0.019	0.023	0.023	−0.003	−0.093*
Hair loss range									
25–49%	−0.107*	−0.052	−0.022	−0.038	−0.048	−0.027	−0.049	−0.061	0.226**
≥50%	−0.082	−0.061	−0.090	0.024	−0.015	−0.078	−0.118*	−0.051	0.269**
Alopecia type									
Multi	0.055	−0.033	−0.027	0.050	−0.013	−0.055	0.017	0.009	−0.062
Ophiasis	0.013	−0.072	−0.015	−0.004	−0.018	−0.091*	−0.044	−0.004	0.035
Others^a^	0.067	0.033	0.083	−0.019	0.038	−0.029	0.050	0.037	−0.057
Relapse experience									
≥1 time	−0.072	−0.036	−0.008	0.028	0.015	0.069	0.019	0.026	0.800
Disease duration									
1–4 years	−0.131**	−0.143**	−0.062	−0.053	0.032	−0.016	−0.138**	−0.012	0.039
≥5 years	0.020	0.044	−0.020	−0.037	0.000	0.050	0.037	−0.022	−0.057
Comorbidities									
With	−0.104*	−0.116**	−0.069	−0.179**	−0.087*	−0.113**	−0.116**	−0.068*	0.083*
Current treatment at hospital									
Yes	0.039	−0.019	0.085	0.003	−0.010	−0.037	−0.038	−0.046	0.195**
Current usage of wigs									
Yes	−0.037	0.019	0.003	0.023	−0.008	0.081	−0.005	−0.018	0.062
HADS‐A	−0.228**	−0.205**	−0.296**	−0.227**	−0.155**	−0.295**	−0.258**	−0.419**	0.094
HADS‐D	−0.246**	−0.382**	−0.204**	−0.400**	−0.517**	−0.269**	−0.383**	−0.368**	0.319**
(Adjusted *R* ^2^)	0.284**	−0.382**	0.245**	0.383**	0.427**	0.317**	0.446**	0.562**	0.409**

*Note:* *p < 0.05; **p < 0.01 versus male (sex), 20–29 years (age), <25% (hair loss range), single (alopecia type), with (comorbidities), 0–11 months (disease duration), never (relapse experience), no (current treatment at hospital), no (current usage of wigs), non‐cases (HADS‐A), or non‐cases (HADS‐D).

Abbreviations: BP, bodily pain; DLQI, Dermatology Life Quality Index; GH, general health; HADS‐A, Hospital Anxiety and Depression Scale – Anxiety; HADS‐D, Hospital Anxiety and Depression Scale – Depression; MH, mental health; PF, physical functioning; RE, role emotional; RP, role physical; SF‐36v2, Short Form Health Survey 36 Item Version 2.0; SF, social functioning; VT, vitality.

^a^ Population in others section included both alopecia totalis and universalis.

The same analysis for DLQI revealed that hair loss range (standardized regression coefficients, 0.226 for <25% vs 25–49%, 0.269 for <25% vs ≥50%), current treatment at hospital (0.195 for “yes” vs “no”) and HADS‐D scores (0.319) significantly worsen DLQI (*p* < 0.01) (Table 4). Furthermore, age and presence of any comorbidity were also significantly related to DLQI (*p* < 0.01). In contrast to the SF‐36v2 analysis, higher HADS‐A scores did not significantly affect DLQI (*p* = 0.107).

## 4 DISCUSSION

This is the first study that comprehensively evaluated the impact of AA on HRQoL in a large Japanese population in comparison to the national standard HRQoL. Patients with AA reported lower HRQoL scores, as evaluated by SF‐36v2, in comparison with Japanese population norms (national standard values for Japanese). This study concluded that HRQoL impairment in patients with AA occurred especially in the mental components with no sex preponderance, indicating worsened SF, higher psychological distress, and diminished energy levels due to the disorder. The study also revealed that more than half of the population were suffering from psychological ailments like anxiety and depression.

Among the eight subscales of SF‐36v2, MH, SF, and VT in patients with AA were relatively lower. A lower MH score reflects that the mental well‐being of patients with AA is significantly disturbed due to disease severity.^26^ Similarly, lower VT scores establish that tiredness and decrease in PF are caused by AA.^26^ Also, lower SF scores demonstrate worsened social relationships of patients with AA and their families and/or other peer groups.^26^ Therefore, in clinical practice, it is imperative to offer counseling for patients with AA, presuming that the patients may be in a weakened mental state. Additionally, when evaluated as two components, mental (MCS) and physical (PCS), a similar tendency was observed as that for the eight subscales, indicating that the mental domains had a large effect, while physical domains had a modest effect. Endo *et al*. reported that in their study subpopulation of AA patients (n = 46) the mean score of MCS for SF‐8 was much lower when compared with scores in the Japanese population (41.6 vs 50.1).^27^ Although they used SF‐8 scale which was known to have lower precision than SF‐36,^28^ the result was quite consistent with our results using SF‐36v2. These results are in line with a study that evaluated SF‐36v2 in Japanese AD patients.^29^ Further, a recent review highlighted that several studies established a strong correlation of AA with the mental components of HRQoL.^6^

The DLQI is a more sensitive assessment tool for evaluating HRQoL of patients with AA, as it is specific for skin diseases.^30^ Multivariate linear regression analysis revealed that hair loss range (<25% vs 25–49% or <25% vs ≥50%), current treatment at hospital (“yes” vs “no”) and depression were the independent factors that most affected DLQI (*p* < 0.01). Several studies have confirmed that DLQI scores increased with the severity of disease. Hence, to improve the HRQoL of patients with AA, it is important to maintain a hair loss range of less than 25% as a recommended treatment threshold. Qualitative interviews conducted with dermatologists and patients with AA suggested that achieving 20% or less scalp hair loss indicated treatment success for patients with 50% or more scalp hair loss.^31^ Our results are in alignment with the aforementioned studies. The additional DLQI outcome on treatment at hospital is as expected because patients with low HRQoL tend to visit the hospital more frequently. The results of depression highlighted the need for psychological intervention for management of AA. For instance, Willemsen *et al*. evaluated hypnotic approaches for AA patients and concluded that hypnosis in refractory AA significantly improves depression, anxiety, and life quality.^32^

Despite differing background demographics and disease severities assessed in other studies, the outcomes (SF‐36v2 and DLQI) of our study were comparable to the study results from other countries (Table S2). The DLQI score (4.8) was similar or slightly better for patients with AA in Japan when compared to the DLQI scores in other countries (5.8–13.5).^6^, ^33^, ^34^ Similar trends were seen when we compared our result with other Japanese studies which evaluated dermatological diseases, namely atopic dermatitis (two papers; DLQI scores, 6.1 and 7.8), urticaria (one paper; DLQI score, 4.8), and psoriasis (three papers; DLQI scores, 4.5, 4.8, and 5.7).^35^, ^36^, ^37^, ^38^ Liu *et al*. conducted a systematic review and concluded that “HRQoL experienced by patients with AA is similar to that seen in patients with other chronic skin diseases including atopic dermatitis and psoriasis.”^8^ Although further evidence is required, current knowledge highlights that the pattern of disease burden in AA patients is common among countries.

The HADS‐A scores in this study indicated that the anxiety score was highest for the hair loss range of 25–49% as compared to the hair loss ranges of less than 25% or 50% or more. It could be interpreted that patients with very severe AA (≥50% hair loss) may cope with their condition by using wigs, with resignation of spontaneous recovery. However, the above‐mentioned pattern was not observed with HADS‐D scores, indicating that anxiety and depression are quite different diseases. Although not completely understood, the relationship between AA and psychological disorders may be bidirectional as stress and anxiety are hypothesized to potentiate AA in some patients.^39^, ^40^

In this study, HRQoL was evaluated using multiple indicators (SF‐36v2, DLQI, and HADS) that consistently reported a decrease in mental HRQoL. This consistency is clinically meaningful and emphasizes the importance of management of the patient’s mental status during routine clinical practice. Further, the mental health scales of SF‐36v2 (MH, SF, RE, VT, and GH) were highly correlated with HADS, as expected. Since SF‐36v2 is a comprehensive questionnaire with 36 questions, it may be difficult and too extensive for some patients. Hence, at an early stage of the disease, it may be recommended to diagnose the decrease in mental HRQoL of patients with AA by using HADS, which has comparatively fewer questions.

Despite the insights gained from this study, there are a few limitations that are worth consideration. In this survey, the study population had minimal racial and cultural bias, as the respondents were enrolled in a self‐selected sample design which may not reflect the entire population. The majority (83.5%) of patients in the survey reported a hair loss range of less than 25%, indicating a limited number of study participants with moderate to severe AA. Furthermore, patient‐reported outcomes are subjective and may involve a risk of recall bias, hence some outcomes might not be accurate from a medical perspective. Additionally, data on the population aged less than 20 years were lacking due to the inability to recruit any patients in the 17–19 years age group. In conclusion, the results of this survey demonstrated that most HRQoL scores of patients with AA evaluated by SF‐36v2 were substantially lower than the Japanese population norms (national standard values for Japanese). HRQoL assessment based on all three assessment tools suggested strong correlation between the severity of AA and lower HRQoL, specifically related to mental health, thus highlighting the importance of understanding the mental health status of patients with AA and the use of psychological interventions as an important tool for the management of patients with AA.

## CONFLICT OF INTEREST

T.I. and S.N. received honoraria for consultancy from Pfizer Japan Inc. K.K., A.Y., and F.M. are employees of Pfizer Japan Inc. and hold stocks or stock options from Pfizer Inc. Y.H. and M.O. are employees of IQVIA Solutions Japan K.K. IQVIA Solutions Japan K.K. was paid for conducting the study and medical writing support from Pfizer Japan Inc.

## Supporting information


Appendix S1

## References

[1] Ito T , Meyer KC , Ito N , Paus R . Immune privilege and the skin. Curr Dir Autoimmun. 2008;10:27–52. 18460879 10.1159/000131412

[2] Paus R , Nickoloff BJ , Ito T . A ‘hairy’ privilege. Trends Immunol. 2005;26:32–40. 15629407 10.1016/j.it.2004.09.014

[3] Villasante Fricke AC , Miteva M . Epidemiology and burden of alopecia areata: a systematic review. Clin Cosmet Investig Dermatol. 2015;8:397–403. 10.2147/CCID.S53985 PMC4521674 26244028

[4] Messenger A , McKillop J , Farrant P , McDonagh A , Sladden M , Hughes J , et al. British Association of Dermatologists’ guidelines for the management of alopecia areata 2012. Br J Dermatol. 2012;166:916–26. 22524397 10.1111/j.1365-2133.2012.10955.x

[5] Ito T . Advances in the management of alopecia areata. J Dermatol. 2012;39:11–7. 22211297 10.1111/j.1346-8138.2011.01476.x

[6] Mostaghimi A , Napatalung L , Sikirica V , Winnette R , Xenakis J , Zwillich SH , et al. Patient perspectives of the social, emotional and functional impact of alopecia areata: a systematic literature review. Dermatol Ther (Heidelb). 2021;11:867–83. 33770385 10.1007/s13555-021-00512-0 PMC8163950

[7] Rencz F , Gulacsi L , Pentek M , Wikonkal N , Baji P , Brodszky V . Alopecia areata and health‐related quality of life: a systematic review and meta‐analysis. Br J Dermatol. 2016;175:561–71. 26914830 10.1111/bjd.14497

[8] Liu LY , King BA , Craiglow BG . Health‐related quality of life (HRQoL) among patients with alopecia areata (AA): a systematic review. J Am Acad Dermatol. 2016;75:806–12 e3. 27436156 10.1016/j.jaad.2016.04.035

[9] Askin O , Koyuncu Z , Serdaroglu S . Association of alopecia with self‐esteem in children and adolescents. Int J Adolesc Med Health. 2020. 10.1515/ijamh-2020-0100 32829314

[10] Senna M , Ko J , Tosti A , Edson‐Heredia E , Fenske DC , Ellinwood AK , et al. Alopecia areata treatment patterns, healthcare resource utilization, and comorbidities in the US population using insurance claims. Adv Ther. 2021;38:4646–58. 34292518 10.1007/s12325-021-01845-0 PMC8408067

[11] Lee S , Lee H , Lee CH , Lee WS . Comorbidities in alopecia areata: a systematic review and meta‐analysis. J Am Acad Dermatol. 2019;80:466–77 e16. 30031145 10.1016/j.jaad.2018.07.013

[12] Gupta MA , Gupta AK . Depression and suicidal ideation in dermatology patients with acne, alopecia areata, atopic dermatitis and psoriasis. Br J Dermatol. 1998;139:846–50. 9892952 10.1046/j.1365-2133.1998.02511.x

[13] Okhovat JP , Marks DH , Manatis‐Lornell A , Hagigeorges D , Locascio JJ , Senna MM . Association between alopecia areata, anxiety, and depression: a systematic review and meta‐analysis. J Am Acad Dermatol. 2019. 10.1016/j.jaad.2019.05.086 31163237

[14] Bilgic O , Bilgic A , Bahali K , Bahali AG , Gurkan A , Yilmaz S . Psychiatric symptomatology and health‐related quality of life in children and adolescents with alopecia areata. J Eur Acad Dermatol Venereol. 2014;28:1463–8. 24237476 10.1111/jdv.12315

[15] Tzur Bitan D , Berzin D , Kridin K , Cohen A . The association between alopecia areata and anxiety, depression, schizophrenia, and bipolar disorder: a population‐based study. Arch Dermatol Res. 2021. 10.1007/s00403-021-02247-6 34089375

[16] Fukuhara S , Bito S , Green J , Hsiao A , Kurokawa K . Translation, adaptation, and validation of the SF‐36 health survey for use in Japan. J Clin Epidemiol. 1998;51:1037–44. 9817121 10.1016/s0895-4356(98)00095-x

[17] Ware JE Jr , Sherbourne CD . The MOS 36‐item short‐form health survey (SF‐36). I. Conceptual framework and item selection. Med Care. 1992;30:473–83. 1593914

[18] Finlay AY , Khan G . Dermatology life quality index (DLQI)—a simple practical measure for routine clinical use. Clin Exp Dermatol. 1994;19:210–6. 8033378 10.1111/j.1365-2230.1994.tb01167.x

[19] Chernyshov PV , Tomas‐Aragones L , Finlay AY , Manolache L , Marron SE , Sampogna F , et al. Quality of life measurement in alopecia areata. Position statement of the European academy of dermatology and venereology task force on quality of life and patient oriented outcomes. J Eur Acad Dermatol Venereol. 2021;35:1614–21. 34107093 10.1111/jdv.17370

[20] Jiang R , Janssen MFB , Pickard AS . US population norms for the EQ‐5D‐5L and comparison of norms from face‐to‐face and online samples. Qual Life Res. 2021;30:803–16. 33025373 10.1007/s11136-020-02650-y PMC7952367

[21] Güleç AT , Tanriverdi N , Dürü C , Saray Y , Akçali C . The role of psychological factors in alopecia areata and the impact of the disease on the quality of life. Int J Dermatol. 2004;43:352–6. 15117365 10.1111/j.1365-4632.2004.02028.x

[22] Janković S , Perić J , Maksimović N , Ćirković A , Marinković J , Janković J , et al. Quality of life in patients with alopecia areata: a hospital‐based cross‐sectional study. J Eur Acad Dermatol Venereol. 2016;30:840–6. 26660721 10.1111/jdv.13520

[23] Ware JE Jr . SF‐36 health survey update. Spine (Phila Pa 1976). 2000;25:3130–9. 11124729 10.1097/00007632-200012150-00008

[24] Hongbo Y , Thomas CL , Harrison MA , Salek MS , Finlay AY . Translating the science of quality of life into practice: what do dermatology life quality index scores mean? J Invest Dermatol. 2005;125:659–64. 16185263 10.1111/j.0022-202X.2005.23621.x

[25] Zigmond AS , Snaith RP . The hospital anxiety and depression scale. Acta Psychiatr Scand. 1983;67:361–70. 6880820 10.1111/j.1600-0447.1983.tb09716.x

[26] Fukuhara S , Suzukamo Y . Manual of SF‐36v2 Japanese version: iHope International Inc. Kyoto; 2014, 2019.

[27] Endo Y , Miyachi Y , Arakawa A . Development of a disease‐specific instrument to measure quality of life in patients with alopecia areata. Eur J Dermatol. 2012;22:531–6. 22743283 10.1684/ejd.2012.1752

[28] Fukuhara S , Suzukamo Y . Instruments for measuring health‐related quality of life ‐ SF‐8 and SF‐36. J Clin Exp Med. 2005;213:133–6.

[29] Arima K , Gupta S , Gadkari A , Hiragun T , Kono T , Katayama I , et al. Burden of atopic dermatitis in Japanese adults: analysis of data from the 2013 National Health and wellness survey. J Dermatol. 2018;45:390–6. 29388334 10.1111/1346-8138.14218 PMC5947641

[30] Basra M , Fenech R , Gatt R , Salek M , Finlay AY . The dermatology life quality index 1994–2007: a comprehensive review of validation data and clinical results. Br J Dermatol. 2008;159:997–1035. 18795920 10.1111/j.1365-2133.2008.08832.x

[31] Macey J , Kitchen H , Aldhouse NVJ , Burge RT , Edson‐Heredia E , McCollam JS , et al. Dermatologist and patient perceptions of treatment success in alopecia areata and evaluation of clinical outcome assessments in Japan. Dermatol Ther. 2021;11:433–47. 10.1007/s13555-020-00477-6 PMC8019002 33464474

[32] Willemsen R , Haentjens P , Roseeuw D , Vanderlinden J . Hypnosis in refractory alopecia areata significantly improves depression, anxiety, and life quality but not hair regrowth. J Am Acad Dermatol. 2010;62:517–8. 20159323 10.1016/j.jaad.2009.06.029

[33] Qi S , Xu F , Sheng Y , Yang Q . Assessing quality of life in alopecia areata patients in China. Psychol Health Med. 2015;20:97–102. 24628099 10.1080/13548506.2014.894641

[34] Al‐Mutairi N , Eldin ON . Clinical profile and impact on quality of life: seven years experience with patients of alopecia areata. Indian J Dermatol Venereol Leprol. 2011;77:489–93. 21727697 10.4103/0378-6323.82411

[35] Mabuchi T , Yamaoka H , Kojima T , Ikoma N , Akasaka E , Ozawa A . Psoriasis affects patient’s quality of life more seriously in female than in male in Japan. Tokai J Exp Clin Med. 2012;37:84–8. 23032250

[36] Masaki S , Tatsukawa R , Uryu M , Takahara M , Furue M , Ohata C , et al. Treatment satisfaction, willingness to pay and quality of life in Japanese patients with psoriasis. J Dermatol. 2017;44:143–6. 27599656 10.1111/1346-8138.13541

[37] Yano C , Saeki H , Ishiji T , Ishiuji Y , Sato J , Tofuku Y , et al. Impact of disease severity on work productivity and activity impairment in Japanese patients with atopic dermatitis. J Dermatol. 2013;40:736–9. 23834561 10.1111/1346-8138.12220

[38] Itakura A , Tani Y , Kaneko N , Hide M . Impact of chronic urticaria on quality of life and work in Japan: results of a real‐world study. J Dermatol. 2018;45:963–70. 29897137 10.1111/1346-8138.14502 PMC6099381

[39] Dai Y‐X , Tai Y‐H , Chen C‐C , Chang Y‐T , Chen T‐J , Chen M‐H . Bidirectional association between alopecia areata and major depressive disorder among probands and unaffected siblings: a nationwide population‐based study. J Am Acad Dermatol. 2020;82:1131–7. 32007291 10.1016/j.jaad.2019.11.064

[40] Vallerand IA , Lewinson RT , Parsons LM , Hardin J , Haber RM , Lowerison MW , et al. Assessment of a bidirectional association between major depressive disorder and alopecia areata. JAMA Dermatol. 2019;155:475–9. 30649133 10.1001/jamadermatol.2018.4398 PMC6459092

