# Supplementary material for: Health‐related quality of life in patients with alopecia areata: Results of a Japanese survey with norm‐based comparisons
Source: J Dermatol. 2022 Mar 28;49(6):584–93. doi: 10.1111/1346-8138.16364 (PMC9314875; doi:10.1111/1346-8138.16364)
Supplement: Supplementary file 1 — Appendix S1 [file JDE-49-584-s001.docx]

**Supplementary documents**

Supplemental figure 1: SF-36v2 subscale scores for patients with alopecia areata.

Supplemental table 1: Subpopulation analysis for SF-36v2, DLQI and HADS in patients with alopecia areata

Supplemental table 2: Comparison with other reported studies


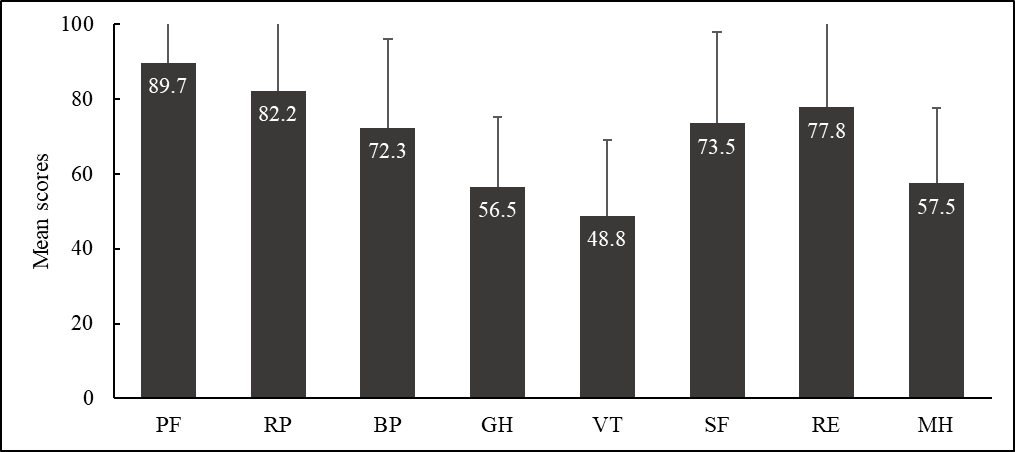


**Supplemental figure 1: SF-36v2 subscale scores for patients with alopecia areata.** Error bars indicate standard deviation. PF: physical functioning; RP: role physical; BP: bodily pain; GH: general health; VT: vitality; SF: social functioning; RE: role emotional; MH: mental health.

## Supplemental table 1: Subpopulation analysis for SF-36v2, DLQI and HADS in patients with alopecia areata

|  | Sex | | Age^†^ | | | | Hair loss range | | | Alopecia type | | | | Comorbidities | |
| --- | --- | --- | --- | --- | --- | --- | --- | --- | --- | --- | --- | --- | --- | --- | --- |
|  | Male | Female | 20-29 | 30-39 | 40-49 | 50-85 | <25% | 25%-49% | ≥50% | Single | Multi | Ophiasis | Others^‡^ | With | Without |
| N | 131 | 269 | 62 | 98 | 129 | 111 | 334 | 33 | 33 | 231 | 136 | 14 | 19 | 155 | 245 |
| SF-36v2^§^, mean (SD) | | | | | | | | | | | | | | | |
| PF | 49.9  (11.0) | 52.2*  (8.0) | 53.9  (5.8) | 51.3*  (9.5) | 51.1*  (9.8) | 50.7*  (9.5) | 52.1  (8.4) | 47.6*  (12.2) | 48.9  (11.6) | 51.0  (9.8) | 52.2  (7.6) | 48.3  (12.0) | 53.5  (7.9) | 49.1  (10.7) | 53.0*  (7.7) |
| RP | 48.1  (11.6) | 48.7  (10.9) | 48.4  (10.7) | 48.5  (10.5) | 47.4  (12.0) | 49.8  (10.7) | 49.1  (10.5) | 44.7  (13.5) | 46.2  (13.3) | 48.6  (11.1) | 48.4  (10.9) | 41.9  (13.7) | 52.7*  (7.9) | 45.2  (12.9) | 50.6*  (9.2) |
| BP | 48.8  (10.3) | 48.3  (10.9) | 51.4  (9.94) | 48.3  (10.7) | 48.4  (10.7) | 47.1*  (11.0) | 48.8  (10.5) | 47.4  (11.4) | 46.7  (12.2) | 48.4  (10.5) | 48.3  (10.7) | 45.2  (13.9) | 53.2  (10.4) | 46.4  (11.2) | 49.8*  (10.2) |
| GH | 48.9  (9.8) | 49.1  (9.6) | 49.5  (8.9) | 48.7  (9.4) | 49.1  (10.0) | 49.0  (9.9) | 49.3  (9.9) | 46.5  (8.2) | 48.8  (7.9) | 49.1  (10.2) | 48.8  (9.3) | 47.1  (8.2) | 51.7*  (4.4) | 45.6  (10.5) | 51.2*  (8.4) |
| VT | 46.9  (9.2) | 45.9  (10.1) | 45.6  (10.1) | 45.5  (9.0) | 46.1  (10.1) | 47.3  (10.1) | 46.5  (9.8) | 43.5  (9.8) | 46.1  (9.5) | 46.0  (10.0) | 46.3  (9.6) | 43.6  (9.3) | 50.2*  (8.2) | 43.8  (10.7) | 47.8*  (8.9) |
| SF | 45.5  (12.0) | 45.9  (10.4) | 45.4  (9.7) | 43.9  (10.9) | 46.2  (11.0) | 47.1  (11.5) | 46.3  (10.6) | 43.4  (10.4) | 42.7  (13.8) | 46.1  (10.8) | 45.7  (10.8) | 38.8*  (12.0) | 47.9  (11.2) | 42.8  (11.7) | 47.6*  (10.0) |
| RE | 47.0  (11.8) | 46.8  (11.6) | 46.0  (12.3) | 46.1  (11.6) | 46.3  (12.4) | 48.7  (10.2) | 47.6  (10.9) | 43.5  (13.9) | 42.6  (14.8) | 46.6  (11.6) | 47.6  (11.5) | 40.9  (12.5) | 50.0  (11.5) | 43.2  (13.3) | 49.2*  (9.7) |
| MH | 45.7  (10.2) | 45.6  (10.1) | 44.5  (10.7) | 44.8  (10.0) | 45.5  (10.3) | 47.3  (9.7) | 46.2  (10.0) | 42.1*  (9.4) | 43.7  (11.1) | 45.4  (10.5) | 46.1  (9.8) | 41.9  (9.6) | 48.4  (8.0) | 43.0  (10.6) | 47.4*  (9.4) |
| DLQI, mean (SD) | 5.5  (5.2) | 4.5  (5.2) | 6.0  (5.8) | 4.9  (4.9) | 5.2  (5.4) | 3.7*  (4.6) | 4.0  (4.2) | 9.3*  (7.2) | 9.1*  (7.1) | 4.6  (5.1) | 4.5  (4.7) | 8.9  (8.8) | 5.7  (5.0) | 6.2  (5.7) | 4.0*  (4.6) |
| HADS, mean (SD) | | | | | | | | | | | | | | | |
| HADS-A | 7.6  (4.4) | 7.3  (4.3) | 8.1  (4.0) | 8.2  (4.3) | 7.4  (4.4) | 6.3*  (4.3) | 7.3  (4.2) | 8.5  (4.7) | 7.3  (4.9) | 7.7  (4.4) | 7.2  (4.3) | 9.4  (3.5) | 4.6*  (2.8) | 8.4  (4.7) | 6.8*  (4.0) |
| HADS-D | 7.3  (4.7) | 6.3*  (4.4) | 6.6  (3.9) | 6.6  (4.4) | 6.9  (5.0) | 6.4  (4.5) | 6.5  (4.5) | 7.3  (4.7) | 7.4  (4.7) | 6.8  (4.7) | 6.4  (4.3) | 7.4  (4.8) | 5.4  (3.7) | 7.5  (4.8) | 6.1*  (4.3) |

## Supplemental table 1: Subpopulation analysis for SF-36v2, DLQI and HADS in patients with alopecia areata (Continued)

|  | Disease duration | | | Relapse experience | | Current treatment at hospital | | Current usage of wigs | | HADS-A^¶^ | | | HADS-D^¶^ | | |
| --- | --- | --- | --- | --- | --- | --- | --- | --- | --- | --- | --- | --- | --- | --- | --- |
|  | 0-11 months | 1-4 yrs. | ≥5 yrs. | Never | ≥1 time | Yes | No | Yes | No | Non | Doubtful | Definite | Non | Doubtful | Definite |
| N | 180 | 112 | 108 | 125 | 275 | 275 | 125 | 22 | 378 | 216 | 84 | 100 | 233 | 89 | 78 |
| SF-36v2^§^, mean (SD) | | | | | | | | | | | | | | | |
| PF | 52.5  (7.2) | 48.8*  (12.3) | 52.6  (7.6) | 53.2  (6.2) | 50.7*  (10.1) | 51.4  (8.8) | 51.7  (9.8) | 50.1  (11.2) | 51.6  (9.0) | 54.6  (3.6) | 51.2*  (9.5) | 44.9*  (13.0) | 54.3  (5.1) | 50.5*  (10.7) | 44.1*  (12.0) |
| RP | 49.4  (10.1) | 45.2*  (12.8) | 50.4  (10.1) | 50.3  (10.0) | 47.7*  (11.5) | 47.7  (11.6) | 50.2*  (9.8) | 48.6  (12.1) | 48.5  (11.0) | 53.1  (6.4) | 46.7*  (11.2) | 40.0*  (13.4) | 52.9  (6.7) | 46.6*  (11.0) | 37.5*  (13.4) |
| BP | 49.3  (10.4) | 47.0  (10.6) | 48.7  (11.3) | 49.5  (10.9) | 48.0  (10.6) | 48.6  (10.6) | 48.1  (11.0) | 48.7  (10.0) | 48.5  (10.8) | 51.9  (9.5) | 47.0*  (9.2) | 42.4*  (11.5) | 51.3  (9.7) | 47.8*  (9.0) | 40.9*  (11.6) |
| GH | 49.6  (9.7) | 48.1  (9.4) | 49.1  (9.9) | 49.6  (10.3) | 48.8  (9.32) | 48.5  (9.3) | 50.3  (10.3) | 49.2  (9.5) | 49.0  (9.6) | 52.8  (8.1) | 47.5*  (7.1) | 42.3*  (10.5) | 52.9  (7.9) | 46.8*  (7.4) | 40.0*  (9.7) |
| VT | 45.9  (9.7) | 46.5  (9.6) | 46.4  (10.2) | 46.7  (10.7) | 46.0  (9.4) | 45.5  (9.8) | 47.9*  (9.6) | 45.4  (8.7) | 46.3  (9.9) | 50.1  (8.1) | 44.7*  (7.7) | 39.2*  (10.6) | 50.4  (7.3) | 44.5*  (8.0) | 35.8*  (10.0) |
| SF | 45.4  (11.0) | 45.0  (11.2) | 47.0  (10.5) | 45.5  (11.9) | 45.9  (10.5) | 44.9  (11.1) | 47.7*  (10.3) | 47.5  (11.6) | 45.7  (10.9) | 49.9  (9.1) | 43.8*  (10.2) | 38.5*  (10.9) | 49.6  (9.1) | 43.2*  (9.7) | 37.1*  (11.5) |
| RE | 47.7  (10.6) | 43.7*  (13.0) | 48.9  (11.1) | 48.0  (11.8) | 46.4  (11.6) | 45.9  (12.1) | 49.1*  (10.2) | 45.1  (12.0) | 47.0  (11.6) | 52.2  (7.1) | 43.9*  (11.6) | 37.9*  (13.1) | 51.7  (7.5) | 43.8*  (12.0) | 36.0*  (12.9) |
| MH | 45.7  (10.1) | 45.3  (10.5) | 46.0  (9.9) | 46.4  (10.5) | 45.3  (9.9) | 44.7  (9.8) | 47.9*  (10.5) | 44.0  (8.0) | 45.8  (10.2) | 51.1  (7.6) | 42.9*  (6.9) | 36.2*  (9.2) | 50.5  (7.5) | 42.8*  (7.9) | 34.6*  (9.3) |
| DLQI, mean (SD) | 4.6  (5.2) | 5.6  (5.7) | 4.3  (4.6) | 3.9  (4.9) | 5.3*  (5.3) | 5.8  (5.6) | 2.6*  (3.4) | 7.9  (5.1) | 4.6*  (5.1) | 3.1  (3.6) | 5.8*  (5.3) | 7.7*  (6.4) | 3.1  (3.7) | 6.2*  (5.3) | 8.3*  (6.5) |
| HADS, mean (SD) | | | | | | | | | | | | | | | |
| HADS-A | 7.6  (4.2) | 7.6  (4.5) | 6.9  (4.3) | 6.7  (4.2) | 7.8*  (4.3) | 7.7  (4.3) | 6.8  (4.4) | 6.8  (4.3) | 7.5  (4.3) | 4.1  (2.1) | 9.0*  (0.8) | 13.3*  (2.0) | 5.2  (3.3) | 9.1*  (3.3) | 12.0*  (3.7) |
| HADS-D | 6.8  (4.5) | 6.7  (4.7) | 6.4  (4.4) | 6.4  (4.7) | 6.7  (4.5) | 7.1  (4.6) | 5.7*  (4.3) | 7.0  (4.8) | 6.6  (4.5) | 4.1  (3.3) | 8.4*  (3.2) | 10.7*  (4.1) | 3.5  (2.3) | 8.9*  (0.8) | 13.5*  (2.6) |

† The study population had no participants with age range between 17 to 19 years.

^‡^ Population in others section included both alopecia totalis and universalis.

* p<0.05 versus male (sex), 20-29 years (age), <25% (hair loss range), single (alopecia type), with (comorbidities), 0-11 months (disease duration), never (relapse experience), no (current treatment at hospital), no (current usage of wigs), non-cases (HADS-A), or non-cases (HADS-D).

**^§^** The scores for SF-36v2 were interpreted using norm-based scoring.

^¶^ Patients were classified into three groups as follows: non-cases (0-7 points for HADS-A or HADS-D score), doubtful cases (8-10 points) and definite cases (11-21 points).

BP: bodily pain; DLQI: Dermatology Life Quality Index; GH: general health; HADS-A: Hospital Anxiety and Depression Scale - Anxiety; HADS-D: Hospital Anxiety and Depression Scale - Depression; MH: mental health; PF: physical functioning; RE: role emotional; RP: role physical; SF: social functioning; SF-36v2: Short Form Health Survey 36 Item Version 2.0; VT: vitality

## Supplemental table 2: Comparison with other reported studies

| **Country** | **Disease** | **N** | **SF-36v2** | | | | | | | | **DLQI** | **Year**  **(Study period)** | **Multivariate**  **analysis** | **Reference** |
| --- | --- | --- | --- | --- | --- | --- | --- | --- | --- | --- | --- | --- | --- | --- |
|  |  |  | PF | RP | BP | GH | VT | SF | RE | MH |  |  |  |  |
| Japan | Alopecia areata | 400 | 89.7 | 82.2 | 72.3 | 56.5 | 48.8 | 73.5 | 77.8 | 57.5 | 4.8 | 2021 | Yes | Current study |
| Serbia | Alopecia areata | 60 | 89.3 | 73.1 | 82.3 | 61.1 | 59.3 | 70.8 | 65.6 | 50.1 | - | 2012-2013 | Yes | 1 |
| Turkey | Alopecia areata | 52 | 90.2 | 83.2 | 76.3 | 65.0 | 51.4 | 71.8 | 59.8 | 55.7 | - | 2001-2002 | Yes | 2 |
| France | Alopecia areata | 60 | 88.2 | 73.3 | 77.2 | 64.3 | 54.5 | 58.9 | 64.1 | 49.3 | - | NA | No | 3 |
| Brazil | Alopecia areata | 37 | 87.5 | 88.5 | 74.3 | 77.9 | 61.8 | 70.6 | 70.3 | 63.9 | - | 2011-2012 | No | 4 |
| Tunisia | Alopecia areata | 50 | 93.1 | 95.5 | 95.4 | 58.2 | 62.4 | 54.6 | 33.3 | 63.6 | - | 2010 | No | 5 |
| Mexico | Alopecia areata | 126 | - | - | - | - | - | - | - | - | 6.1 | 2017-2018 | No | 6 |
| China | Alopecia areata | 698 | - | - | - | - | - | - | - | - | 5.8 | 2010-2012 | No | 7 |
| Iran | Alopecia areata | 176 | - | - | - | - | - | - | - | - | 7.9 | 2013-2014 | Yes | 8 |
| China | Alopecia areata | 130 | - | - | - | - | - | - | - | - | 7.2 | 2013-2014 | No | 9 |
| Iran | Alopecia areata | 100 | - | - | - | - | - | - | - | - | 6.4 | 2009-2010 | No | 10 |
| China | Alopecia areata | 55 | - | - | - | - | - | - | - | - | 8.2 | 2013-2015 | No | 11 |
| Kuwait | Alopecia areata | 2,962 | - | - | - | - | - | - | - | - | 13.5 | 2002-2009 | No | 12 |
| Iran | Alopecia areata | 100 | - | - | - | - | - | - | - | - | 6.3 | 2009-2010 | Yes | 13 |
| USA | Alopecia areata | 532 | - | - | - | - | - | - | - | - | 6.8 | NA | Yes | 14 |
| Japan | Atopic dermatitis | 112 | - | - | - | - | - | - | - | - | 7.8 | 2011-2012 | No | 15 |
| Japan | Atopic dermatitis | 1,668 | - | - | - | - | - | - | - | - | 6.1 | 2017 | No | 16 |
| Japan | Urticaria | 1,443 | - | - | - | - | - | - | - | - | 4.8 | 2017 | No | 16 |
| Japan | Psoriasis | 435 | - | - | - | - | - | - | - | - | 4.8 | 2017 | No | 16 |
| Japan | Psoriasis | 133 | - | - | - | - | - | - | - | - | 5.7 | NA | No | 17 |
| Japan | Psoriasis | 102 | - | - | - | - | - | - | - | - | 4.5 | 2006 | No | 18 |

BP: bodily pain; DLQI: Dermatology Life Quality Index; GH: general health; MH: mental health; NA: not applicable; PF: physical functioning; RE: role emotional; RP: role physical; SF: social functioning; VT: vitality

**Reference**

1 Janković S, Perić J, Maksimović N *et al.* Quality of life in patients with alopecia areata: a hospital-based cross-sectional study. *J Eur Acad Dermatol Venereol* 2016; **30**: 840-6.

2 Güleç AT, Tanriverdi N, Dürü C *et al.* The role of psychological factors in alopecia areata and the impact of the disease on the quality of life. *Int J Dermatol* 2004; **43**: 352-6.

3 Dubois M, Baumstarck-Barrau K, Gaudy-Marqueste C *et al.* Quality of life in alopecia areata: a study of 60 cases. *J Invest Dermatol* 2010; **130**: 2830-3.

4 de Hollanda TR, Sodré CT, Brasil MA *et al.* Quality of life in alopecia areata: a case-control study. *Int J Trichol* 2014; **6**: 8-12.

5 Masmoudi J, Sellami R, Ouali U *et al.* Quality of life in alopecia areata: a sample of tunisian patients. *Dermatol Res Pract* 2013; **2013**: 983804.

6 Velez-Muniz RDC, Peralta-Pedrero ML, Jurado-Santa Cruz F *et al.* Psychological Profile and Quality of Life of Patients with Alopecia Areata. *Skin Appendage Disord* 2019; **5**: 293-8.

7 Qi S, Xu F, Sheng Y *et al.* Assessing quality of life in Alopecia areata patients in China. *Psychol Health Med* 2015; **20**: 97-102.

8 Abedini R, Hallaji Z, Lajevardi V *et al.* Quality of life in mild and severe alopecia areata patients. *Int J Womens Dermatol* 2018; **4**: 91-4.

9 Yu NL, Tan H, Song ZQ *et al.* Illness perception in patients with androgenetic alopecia and alopecia areata in China. *J Psychosom Res* 2016; **86**: 1-6.

10 Ghajarzadeh M, Ghiasi M, Kheirkhah S. Depression and quality of life in Iranian patients with Alopecia Areata. *Iran J Dermatol* 2011; **14**: 140-3.

11 Zhang M, Zhang N. Quality of life assessment in patients with alopecia areata and androgenetic alopecia in the People's Republic of China. *Patient Prefer Adherence* 2017; **11**: 151-5.

12 Al-Mutairi N, Eldin ON. Clinical profile and impact on quality of life: seven years experience with patients of alopecia areata. *Indian J Dermatol Venereol Leprol* 2011; **77**: 489-93.

13 Ghajarzadeh M, Ghiasi M, Kheirkhah S. Associations between skin diseases and quality of life: a comparison of psoriasis, vitiligo, and alopecia areata. *Acta Medica Iranica* 2012; **50**: 511-5.

14 Shi Q, Duvic M, Osei JS *et al.* Health-Related Quality of Life (HRQoL) in alopecia areata patients-a secondary analysis of the National Alopecia Areata Registry Data. *J Investig Dermatol Symp Proc* 2013; **16**: S49-50.

15 Yano C, Saeki H, Ishiji T *et al.* Impact of disease severity on work productivity and activity impairment in Japanese patients with atopic dermatitis. *J Dermatol* 2013; **40**: 736-9.

16 Itakura A, Tani Y, Kaneko N *et al.* Impact of chronic urticaria on quality of life and work in Japan: Results of a real-world study. *J Dermatol* 2018; **45**: 963-70.

17 Masaki S, Tatsukawa R, Uryu M *et al.* Treatment satisfaction, willingness to pay and quality of life in Japanese patients with psoriasis. *J Dermatol* 2017; **44**: 143-6.

18 Mabuchi T, Yamaoka H, Kojima T *et al.* Psoriasis affects patient's quality of life more seriously in female than in male in Japan. *Tokai J Exp Clin Med* 2012; **37**: 84-8.
